# AXL inhibition suppresses early allograft monocyte-to-macrophage differentiation and prolongs allograft survival

**DOI:** 10.1172/jci.insight.178502

**Published:** 2024-01-23

**Authors:** Collin Z. Jordan, Matthew Tunbridge, Irma Husain, Hiroki Kitai, Miriam E. Dilts, Olivia K. Fay, Koki Abe, Catherine Xiang, Jean Kwun, Tomokazu Souma, Edward B. Thorp, Xunrong Luo

**Affiliations:** 1 Division of Nephrology, Department of Medicine, and; 2 Duke Transplant Center, Duke University School of Medicine, Durham, North Carolina, USA.; 3 Adelaide Medical School, University of Adelaide, Adelaide, Australia.; 4 Department of Pathology, Duke University School of Medicine, Durham, North Carolina, USA.; 5 Feinberg Cardiovascular and Renal Research Institute, Department of Medicine, Feinberg School of Medicine, Northwestern University, Chicago, Illinois, USA.

**Keywords:** Immunology, Transplantation, Innate immunity, Macrophages, Organ transplantation

## Abstract

Innate immune cells are important in the initiation and potentiation of alloimmunity in transplantation. Immediately upon organ anastomosis and reperfusion, recipient monocytes enter the graft from circulation and differentiate to inflammatory macrophages to promote allograft inflammation. However, factors that drive their differentiation to inflammatory macrophages are not understood. Here, we show that the receptor tyrosine kinase AXL was a key driver of early intragraft differentiation of recipient infiltrating monocytes to inflammatory macrophages in the presence of allogeneic stimulation and cell-to-cell contact. In this context, the differentiated inflammatory macrophages were capable of efficient alloantigen presentation and allostimulation of T cells of the indirect pathway. Consequently, early and transient AXL inhibition with the pharmacological inhibitor bemcentinib resulted in a profound reduction of initial allograft inflammation and a significant prolongation of allograft survival in a murine heart transplant model. Our results support further investigation of AXL inhibition as part of an induction regimen for transplantation.

## Abstract 2

<p>Receptor tyrosine kinase AXL drives of early differentiation of allograft-infiltrating monocytes to inflammatory macrophages, and AXL inhibition prolongs allograft survival.</p>

## Introduction

Innate immune cells have increasingly recognized roles in solid organ transplantation. In transplantation, the donor organ is first anastomosed to the recipient circulation. Immediately upon anastomosis and reperfusion, recipient immune cells enter the donor organ via circulation. We have previously shown that the process of early recipient monocyte (MC) trafficking to and retention in the donor transplanted organ is mediated via CCL8/CCR8 interaction and is dependent on donor macrophages residing in the transplanted donor organ (1). Through bioinformatics analysis, we determined that recipient MCs, once extravasated, differentiate to macrophages (Mϕs), and replace the donor Mϕs as the new tissue resident Mϕs (2). Others have shown that presence of such recipient Mϕs early after transplantation is associated with acute and chronic kidney allograft rejection (3). However, exactly how recipient intragraft MCs differentiate to tissue resident Mϕs is unclear.

Bioinformatically, recipient receptor tyrosine kinase AXL correlates with the emergence of tissue resident Mϕs in the transplanted solid organ (2). AXL is a member of the TAM family of efferocytic receptors: Tyro3, AXL, and MerTK (4, 5). TAM receptors are known to be expressed by phagocytes and their canonical role is thought to mediate effective clearance of apoptotic cells while inhibiting the associated inflammatory responses during this clearance (5, 6). Therefore, TAM receptors are traditionally associated with inflammation resolution. However, emerging literature suggests that, within the TAM family, individual members may in fact have distinct functions due to their unique ligand specificities, levels of induced expression, protease-mediated extracellular cleavage, and other factors (7–9). Indeed, AXL can be induced by proinflammatory stimuli and has been associated with inflammatory responses (6). Independently in oncology, AXL has also been shown to have a prominent role in cell differentiation and proliferation in cancer cells. Therefore, a small molecule inhibitor of AXL, bemcentinib, which selectively binds to the intracellular catalytic kinase domain of AXL, is a licensed therapy currently in multiple Phase II trials for the treatment of breast, lung, and myeloid neoplasms (10–12).

Previously, in a heart transplant model, we showed that intragraft myeloid cell AXL can facilitate donor-specific T cell and vascular smooth muscle cell proliferation, which in turn promotes cardiac allograft rejection and vasculopathy (13). However, the precise mechanism by which AXL promotes donor-specific T cell proliferation is not known. This critical knowledge gap of the role of AXL in transplantation prevents the rational target of AXL to improve transplantation outcomes.

To address this knowledge gap, in the present study, we investigated the role of AXL in recipient MC-to-Mϕ differentiation and their subsequent capacity for donor antigen presentation and allostimulation in the context of allogeneic solid organ transplantation. Our findings are the first to our knowledge to offer new insight into the mechanism by which AXL promotes alloimmunity and how its inhibition by bemcentinib may be exploited to reduce early posttransplant graft inflammation and prolong allograft survival.

## Results

### MC to inflammatory Mϕ differentiation in response to allogeneic stimulation is dependent on AXL.

We have previously demonstrated in a murine allogeneic kidney transplant model that recipient MCs first arrive at the transplanted kidney as Ly6C^+^F4/80^–^ cells in a donor Mϕ-dependent, CCL8/CCR8 axis–dependent manner (1, 2). In this model, pseudotime analysis using single-cell transcriptomics of the kidney allograft revealed that graft-infiltrating recipient MCs quickly differentiated to inflammatory Mϕs (iMϕs), and this process appeared to correlate with a transient upregulation of the receptor tyrosine kinase AXL in these cells (2). However, the precise role of AXL in allostimulated MC to iMϕ differentiation is not clear. Based on our previous observations, we hypothesized that MC-intrinsic AXL is obligatory to this differentiation.

**Figure 1:**
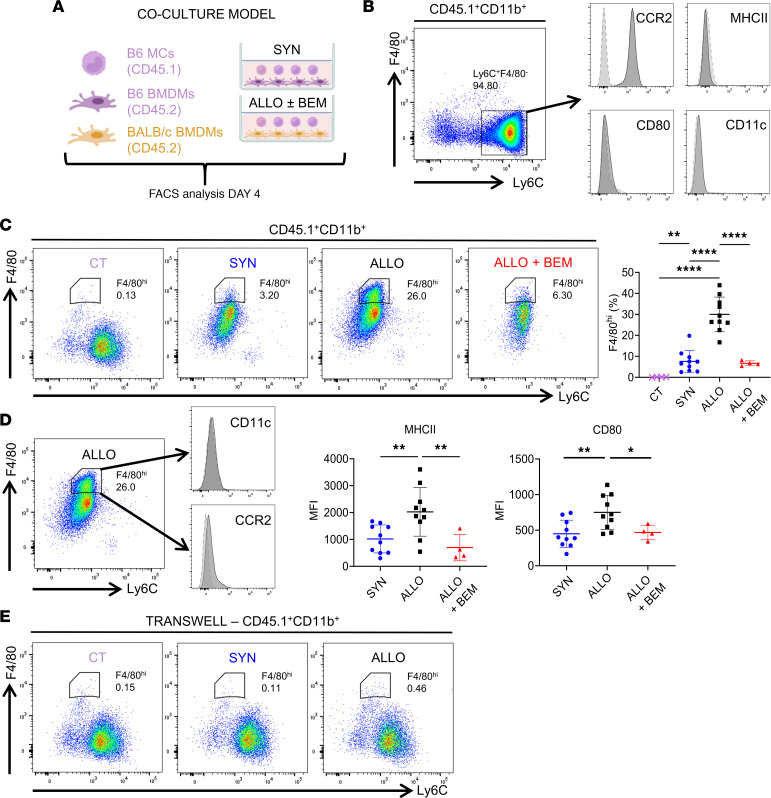
MC to iMϕ differentiation in response to allogeneic stimulation is dependent on AXL. (**A**) Experimental design schematic demonstrating the in vitro allogeneic coculture model. Congenic CD45.1^+^ C57BL6/J (B6) MCs were added into coculture with either CD45.2^+^ syngeneic (B6) or allogenic (BALB/c) BM-derived macrophages (BMDMs). Allogeneic cultures were conducted with or without the AXL inhibitor bemcentinib. FACS analysis was performed after 4 days. (**B**) Representative flow cytometry plots validating the phenotype of B6 CD45.1^+^CD11b^+^Ly6C^+^F4/80^–^ MCs after isolation via magnetic bead–mediated negative selection, demonstrating ~95% purity of the intended population (*n* = 3). (**C**) Representative flow cytometry plots were obtained 4 days after culture of CD45.1^+^CD11b^+^Ly6C^+^ MCs alone (CT), in coculture with syngeneic BMDM (SYN), and in coculture with allogeneic BMDM without bemcentinib (ALLO) or with bemcentinib (ALLO + BEM). Quantification graph of F4/80^hi^ iMϕ frequency under all 4 experimental conditions (*n* = 4–10 per group), analyzed by 1-way ANOVA with corrected post hoc *t* tests. **P < 0.01, ****P < 0.0001. (**D**) Representative flow cytometry plot confirming iMϕ phenotype via absence of CD11c expression and diminishment of CCR2 expression, each respectively compared with an isotype control antibody stain. Normalized mean fluorescence intensity (MFI) of MHCII and CD80 expression gated on iMϕ (*n* = 4–10 per group). Analysis by nonparametric 1-way ANOVA with post hoc Dunnett’s tests. *P < 0.05, **P < 0.01. (**E**) Representative flow cytometry plots obtained on day 4 following culture of MCs alone, and coculture of MCs with syngeneic or allogeneic BMDMs in transwell (plots are representative of *n* = 3 per group).

To test our hypothesis, we set up an in vitro primary coculture system (Figure 1A) in which MCs (Ly6C^+^ cells) isolated from the BM were cocultured with BM-derived Mϕs (BMDMs) for 4 days. The input MCs were CD45.1^+^CD11b^+^Ly6C^+^F4/80^–^CCR2^+^MHC-II^–^CD80^–^CD11c^–^ (Figure 1B). Either syngeneic or allogeneic BMDMs were used. As shown in Figure 1C, MCs cultured alone did not upregulate F4/80. In contrast, MCs cultured with either syngeneic or allogeneic BMDMs significantly upregulated F4/80 while downregulating Ly6C (F4/80^int^Ly6C^lo^). However, only coculturing with allogeneic BMDMs led to the induction of an F4/80^hi^Ly6C^lo^ population (iMϕs). Interestingly, the induction of this population was completely dependent on AXL, since addition of the AXL inhibitor bemcentinib to the coculture effectively blocked its induction. Of note, we determined that it was the MC-intrinsic AXL that was critical for this differentiation by using Axl^–/–^ versus Axl^+/+^ MCs to coculture with allogeneic Axl^+/+^ BMDMs. As shown in Supplemental Figure 1 (supplemental material available online with this article; https://doi.org/10.1172/jci.insight.178502DS1), Axl^+/+^ MCs readily differentiated to F4/80^hi^Ly6C^lo^ iMϕs in the coculture, whereas Axl^–/–^ MCs failed to do so. Of note, Axl^–/–^ mice have no phenotypical abnormalities (2). The differentiated F4/80^hi^ iMϕ population was CD11c^–^ and CCR2^lo/–^, confirming a Mϕ (not a DC) phenotype, and was MHC-II^hi^ and CD80^hi^, supporting their antigen presentation and stimulation ability (Figure 1D). Importantly, in the presence of bemcentinib, MHC-II and CD80 expression by the F4/80^hi^ iMϕs was also significantly inhibited in addition to the inhibition of their development (Figure 1D). To determine if this MC-to-iMϕ differentiation required cell-to-cell contact, we set up the above cocultures using a transwell system, in which MCs and BMDMs were separately placed in the upper and lower chambers. Separation of MCs from BMDMs by transwell completely blocked the upregulation of F4/80 (Figure 1E). Collectively, these data demonstrate that AXL plays a critical role in the process of MC-to-iMϕ differentiation that requires allogeneic stimulation through direct cell-to-cell interactions.

### AXL-dependent MC-to-iMϕ differentiation promotes allospecific T cell proliferation.

**Figure 2:**
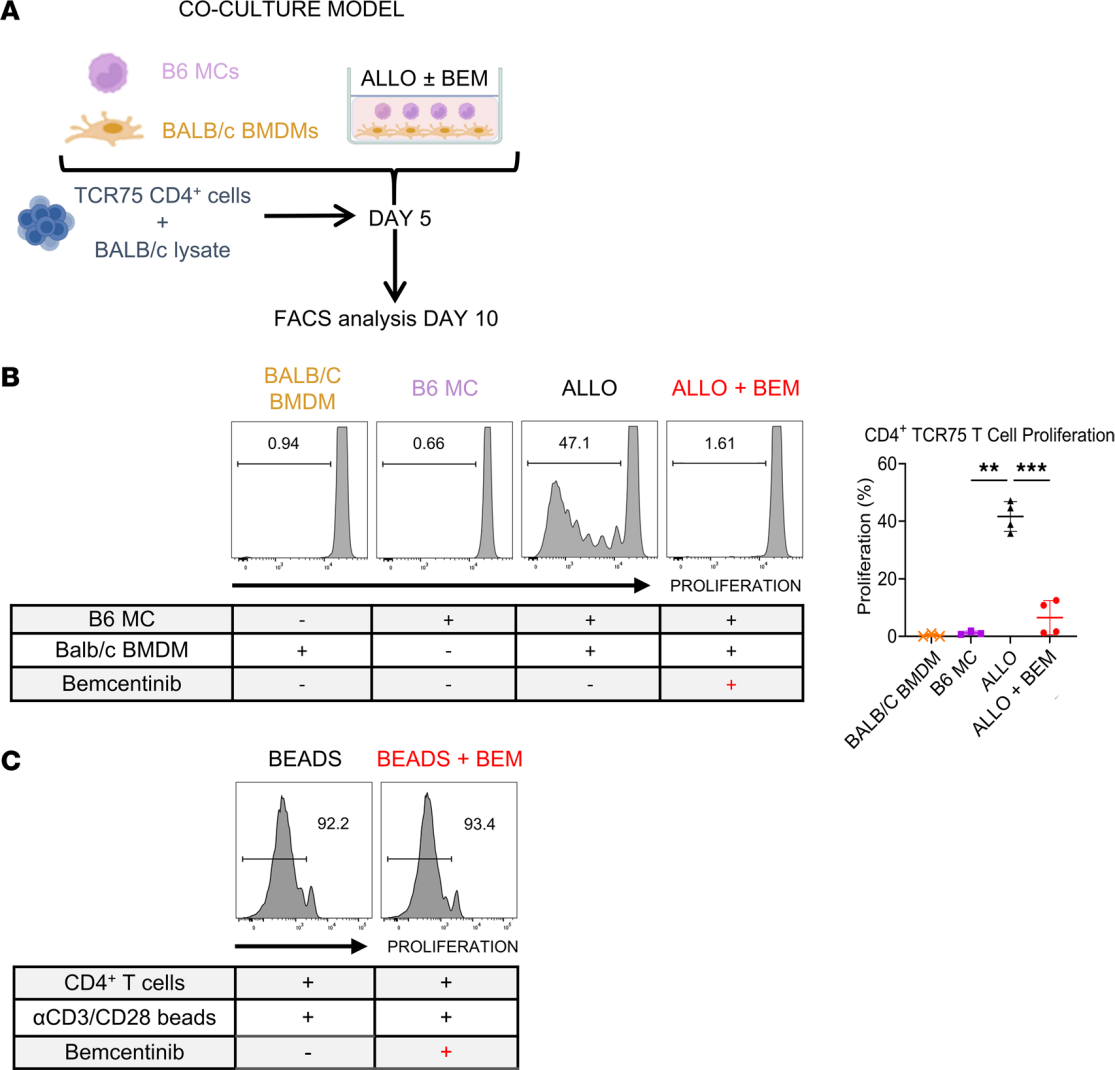
MC-differentiated iMϕs can stimulate allospecific T cell proliferation. (**A**) Experimental design schematic to test the functional capacity of allogeneically differentiated/stimulated iMϕs to induce proliferation of T cells with indirect allospecificity. After 5 days of coculture, V450-labeled B6 CD4^+^ TCR75 transgenic T cells and BALB/c lysate were added. On day 10 of culture, FACS analysis was performed to determine TCR75 T cell proliferation by V450 dilution. (**B**) Representative flow cytometry plots were obtained on day 10 of B6 TCR75 T cells in culture with syngeneic MCs alone (B6 MC), allogeneic BMDMs alone (BALB/c BMDM), or B6 MCs with allogeneic BMDMs without bemcentinib (ALLO) or with bemcentinib (ALLO + BEM). Quantification graph of TCR75 T cell proliferation under 3 experimental conditions for 5 days (*n* = 3–4 per group), analyzed by nonparametric 1-way ANOVA with post hoc Dunnett’s tests. **P < 0.01, ***P < 0.001. (**C**) Representative histograms were obtained on day 5 of B6 CD4 T cells stimulated with anti-CD3/CD28 beads, with or without bemcentinib (*n* = 3).

To determine the role of MC-differentiated iMϕs in instigating and potentiating adaptive alloimmunity, we added C56BL/6J (B6) TCR75 CD4^+^ T cells to the coculture along with BALB/c lysate on day 5 and analyzed TCR75 cell proliferation on day 10 (Figure 2A). TCR75 cells are B6 CD4^+^ T cells that express a transgenic T cell receptor specific for a BALB/c MHC class I peptide presented by B6 MHC class II; they therefore are T cells with indirect specificity in this transplant strain combination (14). As shown in Figure 2B, T cell proliferation did not occur in the presence of either B6 MCs or BALB/c BMDMs alone, and this represented the condition where competent antigen presenting cells (APCs) could not develop in the absence of allogeneic stimulation. The latter represented the condition where there was a complete absence of appropriate APCs capable of stimulating TCR75 T cells. In contrast, robust TCR75 T cell proliferation occurred with the coculture where both B6 MCs and BALB/c BMDMs were present. However, this proliferation was completely inhibited by the addition of bemcentinib to the coculture. Importantly, we ruled out any direct effect of bemcentinib on T cell proliferation, since anti-CD3/28–stimulated T cell proliferation was not affected by the addition of bemcentinib to the culture (Figure 2C).

### Single-cell RNA-Seq analysis of cardiac allografts reveals AXL upregulation during intragraft differentiation of MCs to iMϕs.

**Figure 3:**
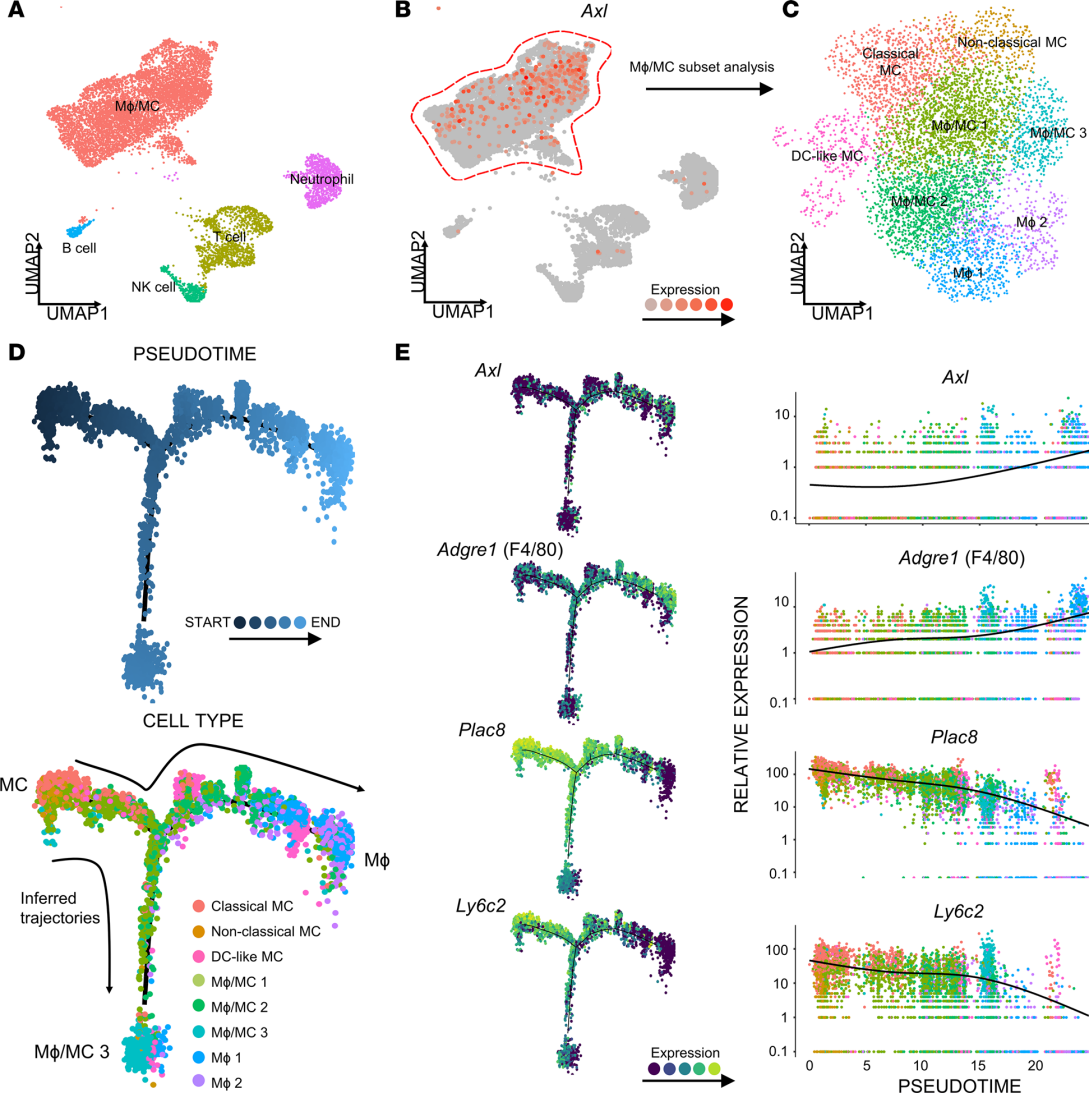
Single-cell RNA-Seq analysis of cardiac allografts reveals AXL upregulation during intragraft differentiation of MCs to iMϕs. (**A**) The single-cell transcriptome map of intragraft immune cell populations (MФ/MC, neutrophils, T cells, B cells, NK cells) at day 5 after transplant in an allogeneic BALB/c to B6 heart transplantation model (*n* = 2). Cells positive for *Ptprc* (CD45) were subset out and displayed in the UMAP plot. Sequencing analysis was performed on a public NCBI data set (GEO accession no. GSE151048). (**B**) UMAP feature plot of the expression of Axl in intragraft immune cell clusters. (**C**) UMAP plot of MФ/MC subcluster analysis (classical MC, nonclassical MC, DC-like MC, MФ/MC 1, MФ/MC 2, MФ/MC 3, MФ 1, and MФ 2). (**D**) Monocle2 trajectory inference analysis of MФ/MC subclusters colored by pseudotime (top trajectory) and cell type (bottom trajectory). (**E**) Expression of *Axl*, *Adgre1* (F4/80), *Plac8*, and *Ly6c2*, as a function of pseudotime (left panels) and cell type (right panels) in Monocle2 trajectory inference analysis.

The above in vitro findings mechanistically explained our previously in vivo observation in a murine allogeneic kidney transplant model (2) that AXL deficiency results in a significant decrease in intragraft Mϕs of the recipient origin. To determine if the AXL-dependent MC-to-iMϕ differentiation could be generalized to other solid organ transplant contexts, we resorted to a publicly available data set of murine cardiac allografts (NCBI; Gene Expression Omnibus [GEO] accession no. GSE151048) (15) and performed an independent single-cell transcriptomic analysis. This data set is composed of 2 BALB/c heart allografts collected from nonimmunosuppressed B6 recipients on day 5 after transplant (15). The sequencing data obtained was analyzed via Seurat, with intragraft immune cells segregated into 5 clusters based on differential gene expressions (Figure 3A and Supplemental Figure 2A). The largest immune cell cluster was Mϕ and MCs (Mϕ/MCs), and the other 4 clusters were T cells, neutrophils, NK cells, and B cells in a descending order of prevalence. As shown in Figure 3B, Axl was almost exclusively expressed in the Mϕ/MC cluster.

Further analysis of this cluster revealed 8 unique subclusters (Figure 3C and Supplemental Figure 2B): classical MCs, nonclassical MCs, DC-like MCs, 3 transitional Mϕ/MC populations (populations 1, 2, and 3), and 2 distinct Mϕ populations (populations 1 and 2). Monocle pseudotime trajectory analysis performed on these clusters revealed an intragraft myeloid cell differentiation pathway from MCs to Mϕs similar to that observed in our murine kidney transplant model (2). Specifically, as shown in Figure 3D, the main trajectory of pseudotime began at the MC node and ended at the Mϕ 1 and 2 nodes, with a separate trajectory to the Mϕ/MC 2 and 3 transitional populations. The other 3 clusters (nonclassical MCs, DC-like MCs, and Mϕ/MC 1) represented additional transitional populations along the main pseudotime trajectory. Importantly, as shown in Figure 3E, Axl was upregulated during the course of MC-to-Mϕ differentiation. This upregulation was parallel to an upregulation of the canonical Mϕ marker Adgre1 (F4/80) and downregulation of the canonical MC markers Plac8 and Ly6c2. Analysis from this independent data set collaterally supports the notion that AXL plays a critical role in intragraft MC-to-Mϕ differentiation.

### Transient AXL inhibition leads to a reduction in early allograft inflammation.

**Figure 4:**
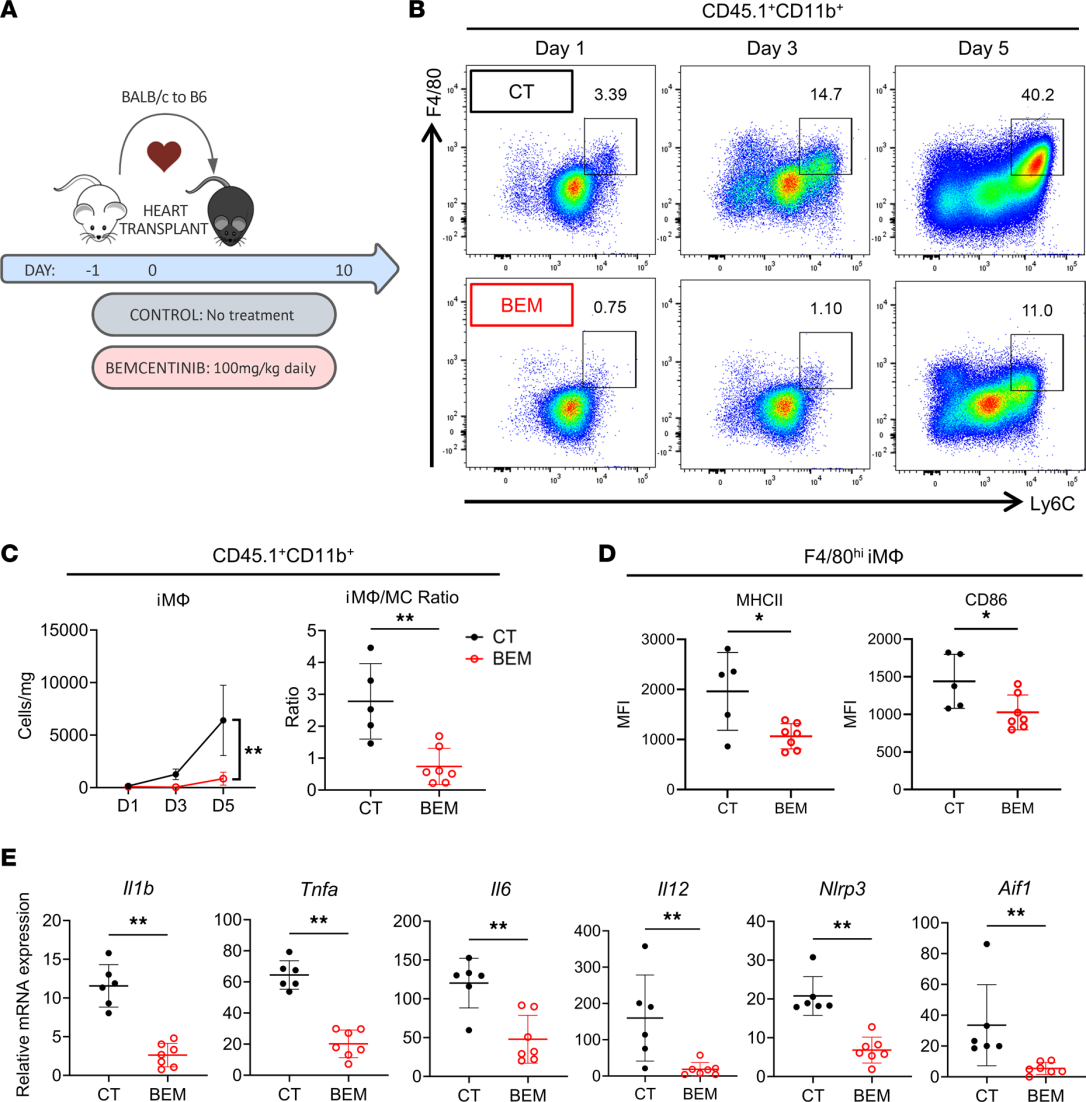
Transient AXL inhibition leads to a reduction in early allograft inflammation. (**A**) Experimental design schematic of a mouse heterotopic heart transplantation model, in which congenic (CD45.1^+^) B6 mice were transplanted with a BALB/c (CD45.2^+^) heart allograft. Control (CT) recipients received no additional treatment, while recipients in the bemcentinib (BEM) group were treated beginning 1 day prior to transplant until 10 days after transplant or until the predetermined sacrificial time point. (**B**) Representative flow cytometry plots of recipient heart allograft myeloid cell infiltrate (gated on CD45.1^+^CD11b^+^ cells) from day 1, day 3, and day 5 after transplant of CT and BEM recipients demonstrating intragraft F4/80^hi^Ly6C^+^ iMϕ differentiation kinetics (*n* = 4–7 per group). (**C**) Quantification graphs at day 1, day 3, and day 5 after transplant of CT and BEM recipients enumerating iMϕ (*n* = 4–7 per group). Quantification graph at day 5 after transplant of the ratio of intragraft iMϕs to MCs (*n* = 5–7 per group), analyzed by Student’s *t* test. **P < 0.01. (**D**) Normalized mean fluorescence intensity (MFI) at day 5 after transplant of MHCII and CD86 expression on intragraft iMϕ from CT and BEM recipients (*n* = 5–7 per group), analyzed by Mann-Whitney sum nonparametric test, *P < 0.05. (**E**) Heart allograft whole tissue mRNA expression of *Il1b*, *Tnfa*, *Il6*, *Il12*, *Nlrp3*, and *Aif1* on day 5 after transplant as measured by semiquantitative PCR; normalized to naive donor heart expression of respective transcripts (*n* = 6–7 per group). Analysis by serial Mann-Whitney sum nonparametric testing. **P < 0.01. Nlrp3, NLR pyrin domain-containing protein 3; *Aif1*, allograft inflammatory factor 1.

Based on our coculture results, we investigated the role for AXL inhibition in vivo using a heterotopic mouse heart transplant model from CD45.2^+^ BALB/c donors to CD45.1^+^ B6 recipients. Recipient mice received either no treatment or oral bemcentinib 100 mg/kg from day –1 to day +10 (Figure 4A). As shown in Figure 4, B and C, mice having received a course of bemcentinib peritransplant exhibited a 7-fold reduction (857 ± 612 versus 6402 ± 3340 cells/mg; *P* < 0.01) in the number of intragraft iMϕs (F4/80^hi^Ly6C^+^ cells) by day 5 after transplant. As such, an accumulation of undifferentiated MCs in the bemcentinib group led to an inversion of the intragraft Mϕ/MC ratio by day 5 after transplant in comparison with that in the control group (Figure 4C). Interestingly, consistent with our in vitro findings (Figure 1D), intragraft recipient iMϕs in the bemcentinib-treated group had significantly lower expression levels of MHC class II and CD86 (Figure 4D). However, the same Ly6C downregulation observed in vitro (Figure 1C) was not observed in vivo (Figure 4B), likely representing a higher level of inflammation in vivo in comparison with in vitro cell cultures (16). Lastly, we also examined inflammatory markers in the heart allograft on day 5 after transplant by whole-tissue quantitative PCR (qPCR). As shown in Figure 4E, there was a global reduction in inflammatory markers including *Il1b*, *Tnfa*, *Il6*, *Il12*, *Nlrp3* (NLR and pyrin domain-containing protein 3), and *Aif1* (allograft-inflammatory factor 1) in the bemcentinib group compared with the control group.

### Transient AXL inhibition leads to a reduction in early adaptive alloimmunity.

**Figure 5:**
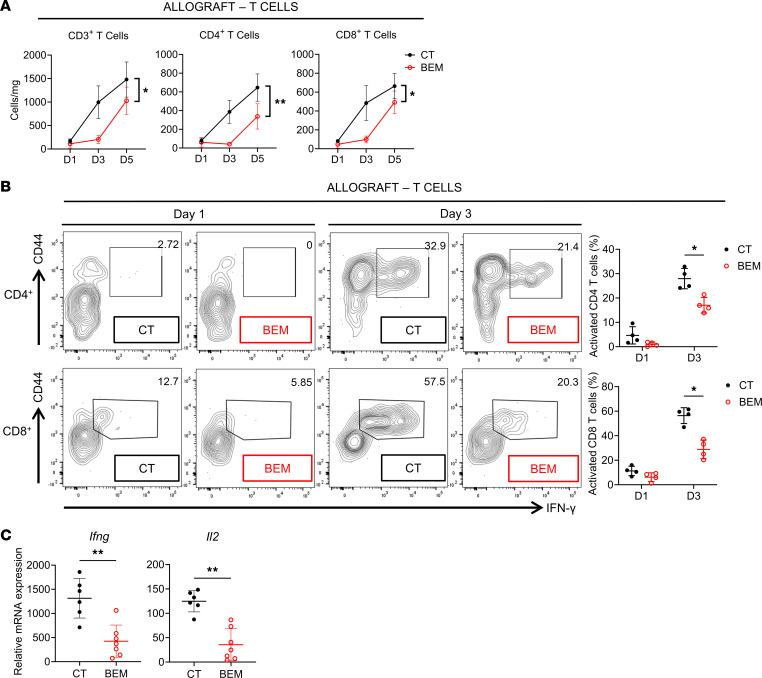
Transient AXL inhibition leads to a reduction in early adaptive alloimmunity. (**A**) Quantification graphs at day 1, day 3, and day 5 after transplant of CT and BEM recipients enumerating recipient intragraft CD3^+^, CD4^+^, and CD8^+^ T cells (*n* = 4–7 per group). (**B**) Representative flow cytometry plots at day 1 and day 3 after transplant of CT and BEM recipients demonstrating intragraft CD4^+^ and CD8^+^ T cell activation and cytokine production as evidenced by CD44 and intracellular IFN-γ expression (*n* = 4 per group). Quantification graph at day 1 and day 3 after transplant of CT and BEM recipients enumerating the percentage of intragraft activated CD4^+^ and CD8^+^ T cells by CD44 and intracellular IFN-γ staining. Analysis for **A** and **B** was done with repeated measures 1-way ANOVA with post hoc *t* tests. *P < 0.05, **P < 0.01. (**C**) Heart allograft whole tissue cytokine profile by mRNA expression of *Ifng* and *Il2* on day 5 after transplant as measured by semiquantitative PCR; normalized to naive donor heart expression of respective transcripts (*n* = 6–7 per group). Analysis by serial Mann-Whitney sum nonparametric testing. **P < 0.01.

We next examined intragraft T cells with or without transient AXL inhibition. We first enumerated graft-infiltrating T cells over time. As shown in Figure 5A, within the cardiac allograft, there was a decrease in recipient CD3^+^ T cells within bemcentinib-treated grafts, both in the CD4^+^ and the CD8^+^ populations. To determine their activation and effector function, we first examined their activation marker CD44 and intracellular IFN-γ production. As shown in Figure 5B, graft-infiltrating CD4^+^ and CD8^+^ T cells from bemcentinib-treated recipients showed a significant reduction of CD44^+^IFN-γ^+^ population on day 3 after transplant in comparison with cells from untreated recipients. Lastly, day 5 whole-tissue allografts were examined by qPCR for expressions of *Ifng* and *Il2*, transcripts canonically associated with T cell activation, and both showed significantly lower levels in bemcentinib-treated recipients in comparison with control untreated recipients (Figure 5C).

### Transient AXL inhibition prolongs murine heart allograft survival.

**Figure 6:**
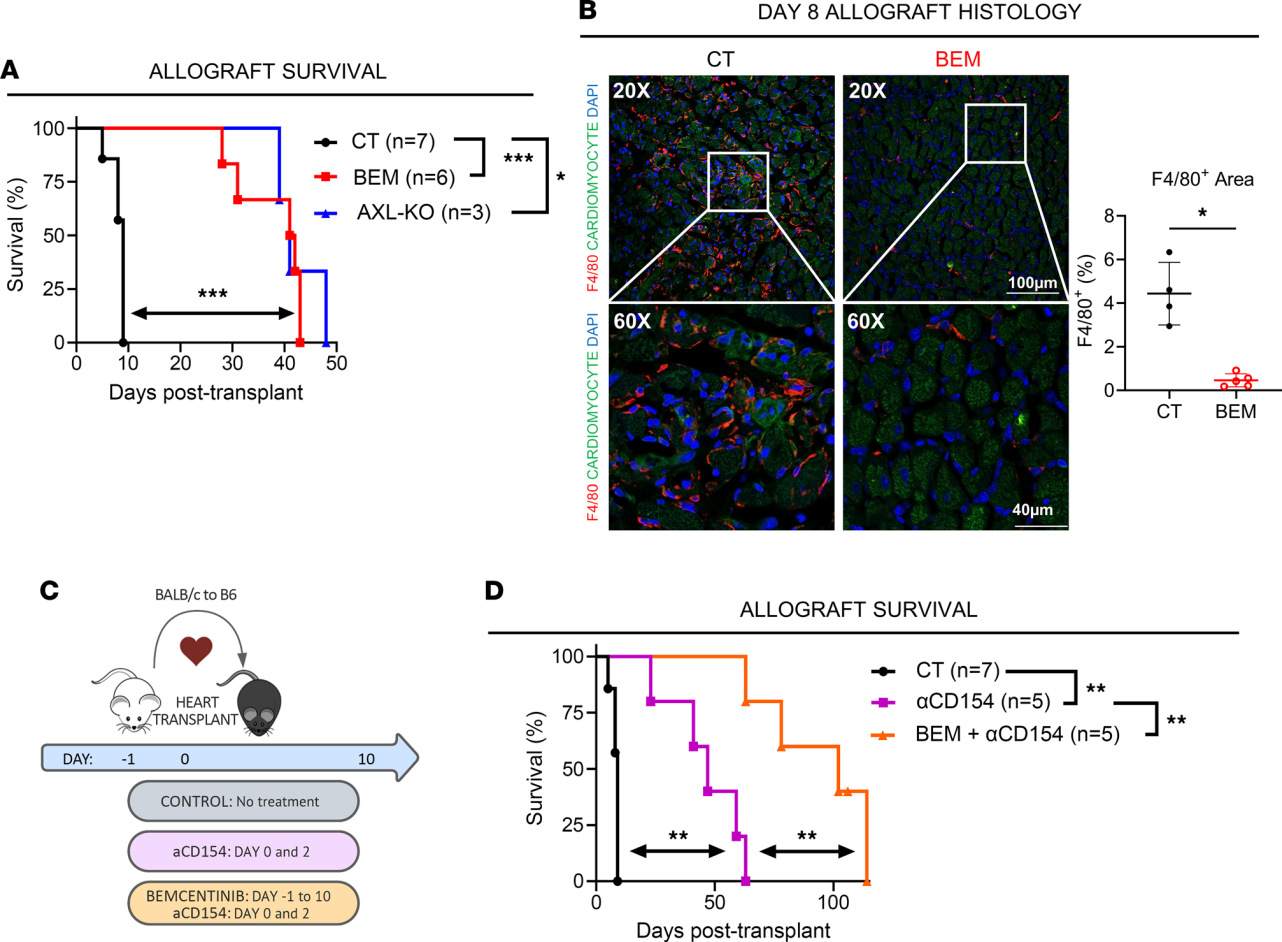
Transient AXL inhibition prolongs murine heart allograft survival. (**A**) Cardiac allograft survival in untreated (CT), transient bemcentinib-treated (BEM) recipients, and AXL-KO mice, plotted as a function of days after transplant with log-rank survival analysis and correction for multiple comparison. *P < 0.05, ***P < 0.001 (*n* = 3–7 per group). (**B**) Representative immunofluorescence imaging of F4/80^+^ Mϕs (red), cardiomyocytes (green, autofluorescence), and nuclei (blue, DAPI) in a heart allograft at time of rejection (day 8) in CT recipients and simultaneous time point of stable graft function in BEM recipients. Scale bar: 100 μm (at ×20), 40 μm (at ×60). Quantification graph of F4/80^+^ Mϕ staining area in heart allograft sections (*n* = 4–5 mice per group; data shown as mean of 4 representative images taken across allograft per recipient). Analysis by *t* test. *P < 0.05. (**C**) Experimental design schematic of a mouse heterotopic heart transplantation model, in which B6 mice were transplanted with a BALB/c heart allograft. CT recipients were left untreated for the duration of the experiment, recipients in the αCD154 group were given 2 doses of perioperative αCD154 (D0 + D2) monotherapy, and recipients in the BEM + αCD154 group were provided transient bemcentinib (D-1 to D+10) and perioperative αCD154 (D0 + D2) dual therapy. (**D**) Heart allograft survival in CT, αCD154 monotherapy, and BEM + αCD154 dual therapy–treated recipients, plotted as a function of days after transplant (*n* = 5–7 per group), with log-rank survival analysis and correction for multiple comparison. **P < 0.01. CT group (*n* = 7) from **A** was used for comparison.

To determine any benefit of transient AXL inhibition and the concomitant reduction of early intragraft inflammation on graft survival, we measured heart allograft survival in our full MHC mismatch heart transplant model. As shown in Figure 6A, bemcentinib monotherapy during the early posttransplantation stage, as in Figure 4A, significantly prolonged cardiac allograft survival, with median survival time (MST) increased from 9 to 41.5 days. Interestingly, transient AXL inhibition from day –1 to +10 had identical graft protective effects as recipient AXL^–/–^ (MST of 43 days; Figure 6A), suggesting that transient AXL blockade is sufficient for the graft protective effect and that, therefore, prolonged AXL inhibition is not necessary. At time of rejection in the control group (day 6–9 after transplant), immunofluorescence staining of F4/80^+^ demonstrated a marked decrease of F4/80^+^ Mϕs in the time-matched heart allografts from the bemcentinib group in comparison with the control group. In addition, heart allografts from the bemcentinib group demonstrated preserved cardiomyocyte viability and architecture in comparison with those from the control group (Figure 6B). Lastly, we combined transient bemcentinib with a short course of CD40-CD154 blockade using anti-CD154 (clone MR1, 250 mg i.p. given on day 0 and day 2; Figure 6C). This combination resulted in an additive graft prolongation to an MST of 102 days (Figure 6D).

Collectively, these data suggest that transient inhibition of AXL posttransplantation significantly reduces alloimmunity and promotes long-term allograft survival, particularly in combination with costimulation blockade.

## Discussion

MCs are key mediators of early posttransplant inflammation (17–20). We have demonstrated the importance of MC-to-Mϕ differentiation in an innate cell in vitro model of alloimmunity. Previous studies suggest that MCs are only partially susceptible to traditional immunosuppression (21). Here, we demonstrate effective blockade of MC-to-Mϕ differentiation by pharmacological inhibition of the AXL pathway, providing an additional target to modulate alloimmunity. In the presence of AXL inhibition by bemcentinib, the resulting Mϕs are not only diminished in number (Figure 1C) but also in their ability to present alloantigen (Figure 1D), with a subsequent reduction in the activation and proliferation of allospecific T cells (Figure 2B). Our monocle psuedotime analysis of a single-cell RNA-Seq data set of murine heart allografts, we observed a similar upregulation of the Axl transcripts during early intragraft MC-to-Mϕ differentiation (Figure 3E), concordant with our published findings in a murine allogeneic kidney transplant model (1). Lastly, transient AXL blockade using bemcentinib treatment in a murine heart transplant model leads to a global reduction in intragraft inflammation and to an extended allograft survival (Figures 4–6).

The AXL signaling pathway is complex. AXL has multiple ligands, the most well recognized of which is Growth arrest signal gene 6 (Gas6). Gas6 is constitutively bound to AXL in tissues by its C-terminal sex hormone–binding globulin (SHBG) domain and canonically acts as a coreceptor for binding target phosphatidylserine moieties at the N-terminal γ-carboxyglutamic acid (GLA) domain (4, 22). Phosphatidylserine is a polarized internal glycerophospholipid component of most cell membranes, and its presence on the cell surface acts as a phagocytic signal (22). Binding of phosphatidylserine to Gas6/AXL causes homodimerization, with autophosphorylation of the intracellular terminal domain and downstream signaling that can result in a broad spectrum of effects (5, 23). AXL upregulation has been shown to promote iMϕs in a range of settings, including postmyocardial infarction (6, 24), nonalcoholic steatohepatitis (25), and renal fibrosis models (26–28). In our model, AXL is a key driver in the pathway of innate cell alloimmunity. It is unclear whether AXL signaling in this context is driven by its canonical ligand-receptor interaction. Innate myeloid cells have the ability to recognize non–self-antigens and become activated in the absence of T cells through pathways such as Signal regulatory protein α (SIRPα)/CD47 interactions (29, 30) and can form innate memory through the paired immunoglobulin-like receptor A (PIR-A)/MHC-I cognate ligand-receptor interaction (31). It is possible that AXL acts through cross-signaling with 1 or more of these pathways, or it may act independently, given its known phagocytic functions. AXL also has the ability to heterodimerize with a range of other receptors in unique conformations (4) that may play a role in non–self-recognition. Regardless of which pathways may be implicated, bemcentinib is a selective small molecule inhibitor of the AXL kinase that inhibits its intracellular autophosphorylation and downstream signaling (10, 32); therefore, it can be exploited to benefit transplant outcome. Importantly, bemcentinib does not affect the function of other TAM kinases that appear to have antiinflammatory effects (4). In particular, MerTK has been found to have a critical role in experimental models of transplant tolerance (33), and these alternative efferocytic pathways may be important modulators of allograft inflammation (6).

Critically, our results show that transient pharmacological AXL inhibition can provide similar benefits to permanent Axl genetic KO (13). In the early posttransplant phase, an inflammatory milieu is potentiated by intragraft Mϕs that release proinflammatory cytokines as well as process and present alloantigens to adaptive immune cells (19, 31, 34). As a key driver of MC-to-Mϕ differentiation, AXL appears to promote Mϕ-driven inflammation in the allograft during this critical period. Therefore, early AXL inhibition during this period results in long-term allograft survival benefit. This is an attractive quality of a transplant therapeutic and raises the potential to further investigate AXL inhibition as part of an induction regimen or during other periods of active allograft inflammation such as with acute rejection. At later stages, Mϕs can promote tissue repair and wound healing, depending on environmental signals (35, 36), and additional benefits may be possible by encouraging polarization toward protissue repair pathways.

In combination with anti-CD154 therapy, bemcentinib at induction showed additive prolongation of allograft survival. Further translational work will need to focus on the optimal role of AXL-targeted therapy in a complete immunosuppression regimen. Nevertheless, AXL is a promising target in organ transplantation, particularly given currently FDA-approved therapeutic options, in contrast to other potential innate cell targeting therapies (37–41). Importantly, bemcentinib is a well-tolerated medication with an acceptable adverse effect profile (11).

Our study is the first to our knowledge to describe a mechanistic role of AXL in intragraft MC-to-Mϕ differentiation and forms a strong foundation for future studies of AXL in transplantation. Immediate future investigations should focus on: (a) the role of AXL on already-differentiated Mϕs in circulation and their entry into the allograft, in addition to the induction of their differentiation in the allograft investigated here; (b) the role of AXL on donor-specific T cell priming in secondary lymphoid organs in addition to the allograft itself; (c) the presence or the absence of tolerogenic features of intragraft Mϕs developed in the absence of AXL, particularly differences between F4/80^int^ and F4/80^hi^ Mϕs, and their respective chemokine productions that may facilitate early infiltration of memory T cells to the graft (42, 43) and/or their proliferation; (d) mechanisms of the ultimate rejection observed in recipients with early AXL inhibition (Figure 6) including whether chronic rejection develops; and (e) whether iMϕs developed in the presence of allogeneic stimulation are cross-decorated (44) and are capable of stimulating the semidirect allorecognition pathway. Ultimately, comparison of single-cell transcriptomics of heart allografts between Axl^+/+^ (15) and Axl^–/–^ recipients will provide a more granular insight to the global downregulation of inflammatory markers shown in Figure 4E in the absence of AXL.

In conclusion, this study shows that AXL is a critical mediator of posttransplant recipient MC-to-Mϕ differentiation and that pharmacological inhibition of its signaling pathway can reduce early allograft inflammation and prolong allograft survival, particularly when in combination with costimulatory blockade.

## Methods

### Sex as a biological variable.

Our current study examined male mice only. Future experiments will extend all of our experiments in this study to female mice. We expect our findings to be relevant to more than 1 sex.

### Mice.

Male BALB/c and C57BL/6J (CD45.1^+^ and CD45.2^+^) mice were purchased from the Jackson Laboratory. All mice were housed at Duke University in a specific pathogen–free facility. All genetically modified mice in this study underwent more than 10 generations of backcrossing.

### Coculture assay.

BM cells were isolated from the tibias and femurs of mice. BMDMs were induced by culturing in RPMI 1640 supplemented with M-CSF (20 ng/mL) for 10 days as previously described (45). Recipient (B6) MCs (CD45.1^+^CD11b^+^Ly6C^+^CCR2^+^F4/80^–^MHCII^–^) were sorted from the BM of CD45.1^+^ B6 mice (Stemcell Technologies) and cultured with either syngeneic or allogeneic donor (BALB/c) CD45.2^+^CD11b^+^ F4/80^+^ BMDM. Purified MCs cultured alone served as a control. In the allogeneic setting, bemcentinib, a selective pharmacological inhibitor of AXL kinase activity (MedChemExpress), was added at a concentration of 1 μM. After 4 days of culture under these 4 conditions, cells were harvested with 0.25% trypsin-EDTA, and surface staining was performed for phenotypic analysis via flow cytometry.

In a separate functional assay, CD4^+^ T cells were isolated from transgenic B6 TCR75 mice. These CD4^+^ T cells express a transgenic T cell receptor that recognizes BALB/c MHC class I molecule H2-K^d^ (peptide sequence 54–68) restricted by B6 MHC class II I-A^b^. TCR75 CD4^+^ cells were stained with V450 cell proliferation dye (Invitrogen) and added to the coculture at day 5 together with BALB/c splenocyte lysate. At day 10 of culture, TCR75 T cells were analyzed by flow cytometry for proliferation.

To determine if there was a direct effect of bemcentinib on T cell proliferation, anti-CD3/CD28 beads (DynaBeads Mouse T Activator, Invitrogen) were used to nonspecifically stimulate T cell proliferation over 5 days in the absence or presence of bemcentinib (1 μM). At day 5 of culture, cells were analyzed as described above.

To determine if the effects of AXL inhibition were due to MC rather than BMDM-specific effects, recipient (B6) MCs (CD45.2^+^CD11b^+^Ly6C^+^CCR2^+^F4/80^–^MHCII^–^) were sorted from the BM of CD45.2^+^ B6 and B6 Axl^–/–^ mice (Stemcell Technologies) and cultured with allogeneic donor (BALB/c) CD45.1^+^CD11b^+^F4/80^+^ BMDM. At day 4 of culture, cells were analyzed as described above.

### Mouse heterotopic cardiac transplantation experiments.

Ten- to 12-week-old CD45.2^+^ BALB/c (H-2^d^) mice were used as donors to either 10- to 12-week-old CD45.1^+^ B6 (H-2^b^) mouse recipients, or B6 AXL-KO mouse recipients. Heterotopic abdominal cardiac transplantation and determination of graft rejection was performed as previously described (46). Mice received either no immunosuppression (control, *n* = 20) or 100 mg/kg bemcentinib by oral gavage daily from day –1 to day +10 after transplantation (BEM group, *n* = 20). In the indicated experiment, mice further received either no immunosuppression (control, *n* = 7), anti-CD154 Bio X Cell MR1 clone 0.25 mg on days 0 and 2 by i.p. injection (αCD154, *n* = 5), or bemcentinib plus anti-CD154 in a combination of the above regimens (BEM/αCD154, *n* = 5). At the rejection or sacrifice time point, cardiac allografts were perfused with PBS and then split for whole-tissue PCR, histology, or flow cytometry.

### RNA extraction and reverse transcription PCR (RT-PCR).

Whole-tissue semiquantitative PCR was conducted on RNA isolated using Trizol Reagent (Invitrogen), after which RNA was converted to cDNA via reverse transcriptase (Verso cDNA synthesis kit, Invitrogen). Semi-quantitative real-time PCR (Applied Biosystems 7500 Real-Time PCR System) was performed in triplicate using TaqMan assay master mix. The following probes were used: *Il1b* (TaqMan Assay ID Mm00434228_m1), *Tnfa* (TaqMan Assay ID Mm00443258), *Il6* (TaqMan Assay ID Mm00446190_m1), *Il12* (TaqMan Assay ID Mm99999067_m1), *Nlrp3* (TaqMan Assay ID Mm00840904_m1), *Aif1* (TaqMan Assay ID Mm00479862_g1), *Il2* (TaqMan Assay ID Mm00434256_m1), and *Ifng* (TaqMan Assay ID Mm01168134_m1). ΔΔCT was used for determination of relative mRNA expression to *Gapdh* (TaqMan Assay ID Mm99999995_g1) and normalized to expression in naive heart samples.

### Immunofluorescence staining and quantification.

Immunofluorescence was performed on samples fixed for 24 hours in paraformaldehyde, transferred to 30% sucrose solution for 24 hours, and then snap-frozen in OCT media at –80°C. Sections were blocked from nonspecific staining using animal-free blocker, 1× in Triton-X100/PBS solution for 45 minutes at room temperature. To evaluate Mϕ infiltration in rejecting cardiac allografts, frozen heart sections were primarily stained using rat anti–mouse F4/80 antibody (1:200 dilution; A3-1, Bio-Rad), incubated overnight at 4°C. Secondary staining was performed using Alexa Fluor 594–conjugated donkey anti–rat IgG (1:400 dilution, Invitrogen) for 2 hours. Finally, nuclei were stained for DAPI (2 μg/mL, Invitrogen) for 10 minutes at room temperature. Sections were then mounted using DAKO Fluorescence Mounting Media and allowed to dry for 15 minutes. Slides were imaged using Carl Zeiss 780 Inverted Confocal Microscopy (ZenBlue 3.7 software), and blinded F4/80^+^ (% area) staining quantification was performed by using NIH ImageJ software.

### Flow cytometry.

Flow cytometry was conducted on coculture samples and cardiac allograft tissue at predetermined time points. Coculture samples were harvested as outlined above before being surface-stained with commercial fluorophore-conjugated antibodies for 40 minutes at 4°C. Following staining incubation, cells were fixed with 4% PFA (Santa Cruz Biotechnology Inc.). Cardiac allograft tissue was digested to a single-cell suspension using collagenase type IV (2 mg/mL, Worthington Biochemical Corporation) with mechanical agitation for 30 minutes at 37°C, followed by RBC lysis with ammonium-chloride-potassium lysis buffer, before staining for extracellular surface markers as per above (1). Prior to staining, cells were incubated for 15 minutes at 4°C with purified anti-mouse Fc shield (anti-CD16/32; 2.4G2, Tonbo Biosciences 70-0161-U100). For intracellular staining, cells were first stimulated with PMA, ionomycin, and brefeldin A (Invitrogen) for 5 hours before surface staining. Following surface staining, cells were fixed with 4% PFA and were then permeabilized (Cytofix/Cytoperm Buffers; BD Biosciences) and stained for IFN-γ for 1 hour at room temperature. Cell characterization data were acquired on a BD Fortessa X20 flow cytometer and analyzed using FlowJo V10.9.0 software. The following antibodies were used: Ly6C-PerCP-Cy5.5 (HK1.4; Invitrogen, 45-5932-82), CD80–Pacific Blue (16-10A1; BioLegend, 104724), CD86-BV605 (GL-1; BD Biosciences, 563055), CCR2-BV711 (475301; BD Biosciences, 747964), MHC-II-BV786 (M5/114.15.2; Invitrogen, 417-5321-82), CD11c-BUV395 (HL3; BD Biosciences, 564080), CD11b-BUV805 (M1/70; BD Biosciences, 741934), CD45.1-APC (A20; Invitrogen, 17-0453-82), F4/80-APCCy7 (BM8; BioLegend, 123118), CD45.2-PECy7 (104; Invitrogen, 25-0454-82), CD3-FITC (17A2; Invitrogen, 11-0032-82), CD4-e450 (GK1.5; Invitrogen, 48-0041-82), CD45.2-BV650 (104; BD Biosciences, 740490), F4/80-APC (BM8; Invitrogen, 17-4801-82), CD44–APC 780 (IM7; Invitrogen, 47-0441-82), CD8a-PE (53-6.7; Invitrogen, 12-0081-82), CD45.1-PECy7 (A20; BioLegend, 110730), CD44-BV786 (IM7; BD Biosciences, 563736), CD4-BUV805 (GK1.5; BD Biosciences, 612900), IFN-γ–APC (XMG1.2; Invitrogen, 17-7311-82), CD8a-PECy7 (53-6.7; Invitrogen, 25-0081-82), Vβ8.3-FITC (1B3.3; BD Biosciences, 553663), CD4-BV650 (GK1.5; BD Biosciences, 565232), CD3–APC 780 (17A2; Invitrogen, 47-0032-82), and CD90.2-PE (30-H12; Invitrogen, 14-0903-82). Dead cells were excluded using Fixable Aqua LIVE/DEAD staining dye (Invitrogen, L34957). FACS analysis was utilized to quantify cell counts per milligram of allograft tissue using the formula:


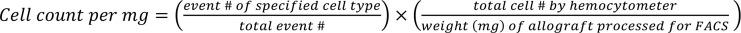


### Single cell RNA-Seq analysis on NCBI.

To analyze the intragraft expression of Axl in a mouse model of allogeneic heart transplantation, raw fastq data were downloaded from public data set NCBI (GEO accession no. GSE151048; sample ID: GSM4565358 and GSM456359) using SRA tool kit (ver.2.9.6-1) (15). Raw fastq data files were aligned to the *mm10* mouse genome reference and quantified using 10x Genomics Cell Ranger (ver.6.0.1). Ambient RNA contamination estimation and removal was performed using R package SoupX (ver.1.6.2) (47). Unique molecular identifier (UMI) counts were then further analyzed using R package Seurat v.4.2.0 for quality control, dimensionality reduction, and cell clustering (48). The single-cell RNA-Seq matrices were filtered by custom cutoffs as follows: genes expressed in < 1 cells, cells expressing < 200 genes, and cells with percentage of mitochondrial genes > 20% were all excluded from further analysis. DoubletFinder (ver.2.03) was then used to remove the potential doublets (49). After removing the ambient RNA and doublets, count matrices from each sample were integrated using Seurat’s integration and label transfer method, which corrects potential batch effects (48, 50). To remove an additional confounding source of variation, the mitochondrial mapping percentage was regressed out. The integrated data set was used for all downstream analyses. A graph-based clustering approach in Seurat was then used to cluster the cells in our integrated data set, with resolution set at 1.5. Cluster-defining markers were obtained using Seurat’s FindAllMarkers command (genes expressed in at least 20% of cells within the cluster, log fold change > 0.25) with the Wilcoxon rank-sum test. Based on the marker genes and manual curation of the gene expression pattern of canonical marker genes in Uniform Manifold Approximation and Projection (UMAP) plots, we assigned a cell identity to each cluster. Cells positive for *Ptprc* (CD45)were assigned as immune cells and displayed on a new subset UMAP plot. To perform trajectory inference analysis on cells within the annotated MФ/MC cluster, a subclustering analysis was used to yield 11 cell types, for which subcluster-defining markers were obtained using Seurat’s FindAllMarkers command. Based on the marker genes and manual curation of the gene expression pattern of such genes in the UMAP plots, a cell identity was assigned to each subcluster. Trajectory analysis was then performed using Monocle 2 (51). Nonnormalized gene expression count data from the Seurat object of the MФ/MC subclusters were used as inputs to create newCellDataSet. Genes for ordering cells were selected if they were expressed in ≥ 10 cells and their mean expression value was ≥ 0.05. Differential gene expression between clusters was calculated using Monocle2’s differentialGeneTest function. Cells were then ordered along a pseudotrajectory using the reduceDimension function (method = ‘DDRTree’) and the orderCells function. The pseudotime trajectory plot was generated using Monocle2’s plot_cell_trajectory function, where cells were defined by cell type or pseudotime placement. *Plac8*, *Ly6c2*, *Adgre1* (F4/80), and *Axl* expression by pseudotime and cell type was plotted using the plot_genes_in_pseudotime function in Monocle2.

### Statistics.

All statistical analysis was conducted using GraphPad Prism version 10.0.0 for Windows, GraphPad Software. Data are presented as mean ± SD for normally distributed data or median ± IQR for nonparametric data. Differences between continuous variables were tested using 1-tailed Student’s *t* test or using Wilcoxon-rank sum test for nonparametric data. Within experiments, correction for multiple comparisons was made using 1-way ANOVA and post hoc multiple comparisons, with repeated measures and nonparametric testing where appropriate. Survival analysis was conducted using log-rank (Mantel-Cox) comparison, with correction for multiple-comparison *P* values. A *P* value less than 0.05 was considered significant.

### Study approval.

This study was approved by the IACUC at Duke University, protocol no. A215-21-10.

### Data availability.

Values for all data points in graphs are reported in the Supporting Data Values file. Single-cell RNA-Seq analysis was conducted on a public NCBI data set (GEO accession no. GSE151048).

## Author contributions

CZJ, EBT, and XL designed the research study. CZJ, MT, EBT, and XL analyzed the data and wrote the manuscript. CZJ, IH, MED, OKF, JK, KA, and CX performed the experiments. HK and TS performed RNA-Seq data analysis. XL supervised the overall project.

## Supplementary Material

Supplemental data

Supporting data values

## Version 1 01/23/2024

In-Press Preview

## Version 2 03/08/2024

Electronic publication
