# Supplementary material for: AXL inhibition suppresses early allograft monocyte-to-macrophage differentiation and prolongs allograft survival
Source: JCI Insight. 2024 Jan 23;9(5):e178502. doi: 10.1172/jci.insight.178502 (PMC10972596; doi:10.1172/jci.insight.178502)
Supplement: Supplemental data [file jciinsight-9-178502-s008.pdf]

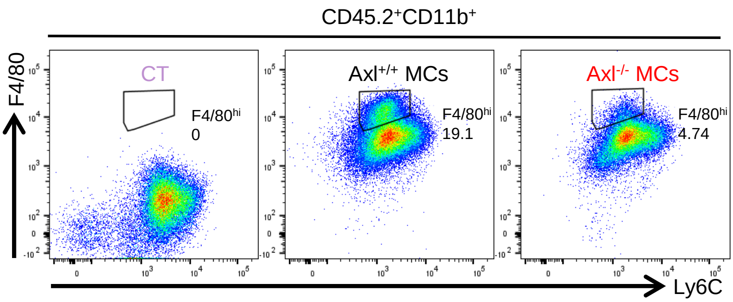

Supplemental Figure 1: MC-intrinsic Axl expression is required for iMϕ differentiation. (A) Representative flow cytometry plots were obtained four days (Day 4) after culture of CD45.2$^+$CD11b$^+$Ly6C$^+$ Axl$^{-/-}$ MCs alone, or co-culture of Axl$^{+/+}$ MCs or Axl$^{-/-}$ MCs with allogeneic Axl$^{+/+}$ BMDMs.

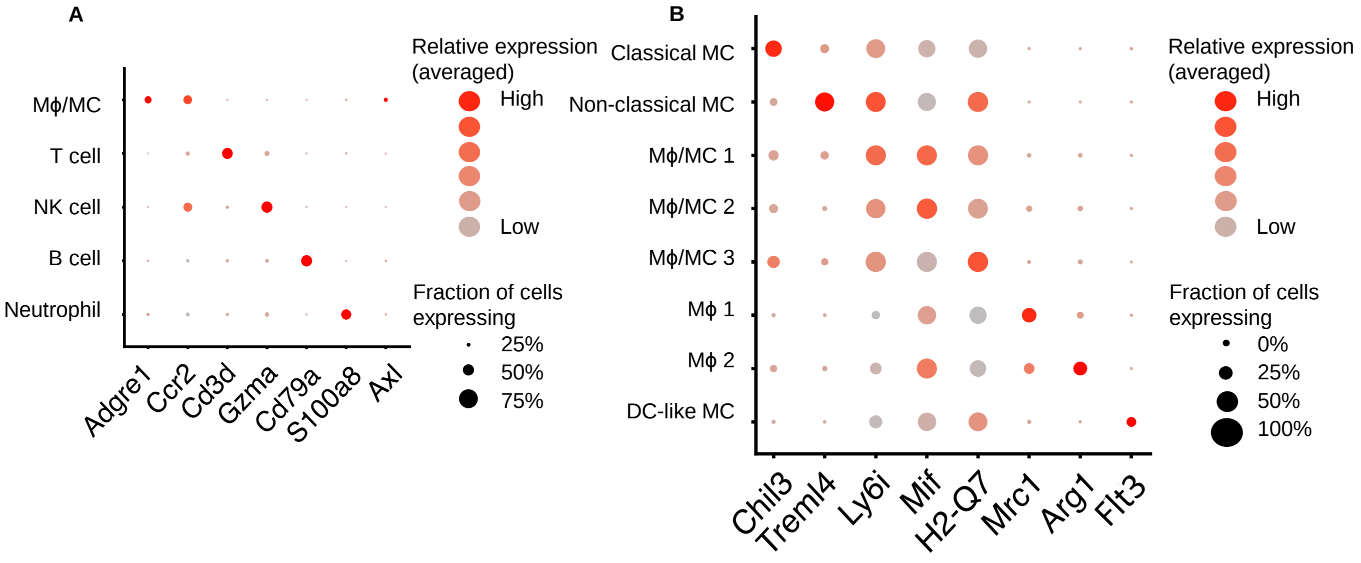

Supplemental Figure 2: Cluster-defining genes of heart allograft UMAP plots. (A) Dot plot demonstrating expression pattern of cluster-enriched canonical marker genes among all *Ptprc* (CD45)-positive cells. **(B)** Dot plot demonstrating expression pattern of Mϕ/MC sub cluster-specific marker genes.
